# Efficacy of Fibrin Glue on Seroma Formation after Breast Surgery

**DOI:** 10.1155/2012/643132

**Published:** 2012-09-12

**Authors:** Mahmood Reza Miri Bonjar, Hemmat Maghsoudi, Roya Samnia, Parviz Saleh, Farhang Parsafar

**Affiliations:** ^1^Department of Surgery, Faculty of Medicine, University of Medical Sciences of Tabriz, Iran; ^2^The Sina Hospital, Azadi Street, P.O. Box 1548, East Azarbaijan, Tabriz 5163639888, Iran

## Abstract

*Background and Objectives*. This study was designed to determine the effectiveness of fibrin glue plus conventional drain placement versus conventional drain placement in the prevention of seromas after breast procedures. Among methods employed to reduce seroma magnitude and duration, fibrin glue has been proposed in numerous studies, with controversial results. *Design and Setting*. A prospective, randomized, controlled study of subjects who were randomized into control and experimental groups was conducted. *Methods*. Collected data included age, surgeon, medical and surgical history, comorbidities, procedure performed, number of axillary nodes, number of positive axillary nodes collected, final pathologic diagnosis, cancer stage, hospital stay, postoperative day of drain removal, complications, incidence of seroma formation, interval to seroma resolution, and number of postoperative visits. *Results*. Analysis of 60 patients showed similarly matched groups. Seroma formation rate was 24.1% in the control group and 16.1% in the fibrin glue group. The rate of wound complications was similar. *Conclusions*. Although use of fibrin sealant resulted in a nonsignificant decrease in seroma formation rate compared with that of drain placement, the higher cost and cumbersome technique tend to indicate that there is no advantage to using fibrin glue over drain placement with the technique described.

## 1. Introduction 

Ever since mastectomy was first carried out by Halsted in 1882, surgeons have faced several problems such as necrosis of the skin flaps, breakdown of the wound, hematoma, seroma, and infection [1]. Among them, seroma, a subcutaneous collection of serous fluid, is a common problem in breast surgery. As it usually resolves within a few weeks, many surgeons view this problem as an unavoidable nuisance rather than a serious complication [1, 2]. However, excessive accumulation will stretch the skin and cause it to sag, resulting in patient discomfort and sometimes prolongation of hospital stay [3]. Of the >180,000 women diagnosed with breast carcinoma in the United States every year, the majority underwent some type of surgical intervention [4]. In 1999, >70% of women in the United States diagnosed with infiltrating breast cancer underwent modified radical mastectomy with axillary dissection. Lumpectomy with axillary dissection is gaining popularity as a breast-conservation option. Sentinel node biopsy has recently come to the forefront as the first line in axillary surveillance. Unfortunately, working in the axillary region as well as the chest wall is fraught with a high incidence of complications including infection, hematoma, wound dehiscence, necrosis of the skin flap, nerve damage, and—most commonly—seroma formation. Many different methodologies have been used in an effort to reduce seroma formation following breast surgery for breast cancer. These include suction drainage [5, 6], topical application of tetracycline, [7] closing and stitching the axillary fossa, [8, 9] axillary dissection with axilloscopy, [10] external compression, [11, 12], and application of fibrin glue [13–15]. To date, no practical guidelines exist on how to conduct suction drainage, and the views of surgeons are varied. Some surgeons believe that it is best to remove the drain on the first day of the postoperative period and some think the drain should be left in place until the drained volume falls below 50 mL/d, [16] while others feel that drainage could in fact prolong and intensify the inflammatory stage of the wound-healing process, with a subsequent increase in seroma formation [17].

Regardless of contradicting opinions, the possibility of reducing the time for which drainage is present during the recovery period, and thus eliminating the need to discharge patients with drainage in situ, may represent a valid solution for reducing seroma formation. The purpose of this study was to evaluate the efficacy of fibrin glue plus closed suction drain over closed suction drain placement in preventing seroma formation after modified radical mastectomy, and lumpectomy with axillary node dissection for the treatment of breast cancer.

## 2. Patient and Methods

A prospective, randomized, controlled, multicenter study was performed between 21 March 2010 and 21 March 2011. Participants were selected from eligible women scheduled for breast procedures at 4 hospitals (Amam Reza, Sina, Shams, and Nejat hospitals). The Human Subjects Committee of the Tabriz University of Medical Sciences approved the study.

Eligible participants (1) were female, (2) were >18 years old, (3) were scheduled to undergo an elective breast procedure (modified radical mastectomy, lumpectomy with axillary node dissection, (4) provided informed consent, (5) did not meet any of the defined exclusion criteria, (6) had platelet counts ≥100,000/mL, (7) have no uncompensated diabetes or advanced liver disease, (8) have no psychological changes, (9) are not severely obese, and (10) have not had previous surgery on the axillary lymphatic system. Potential participants were excluded based on the following criteria: (1) age <18 years, (2) male, (3) steroid use, (4) systemic anticoagulation, (5) ongoing systemic infection at the time of surgery, (6) history of chest irradiation, (7) platelet count ≤99,000/mL, (8) planned immediate breast reconstruction, (9) pregnant or lactating, and (10) hypersensitivity to bovine protein. Short-term antibiotic prophylaxis was applied. 

Patients were randomized into the experimental (fibrin glue plus Jackson-Pratt drain, 31 patients) and control (Jackson-Pratt drain, 29 patients) group. Participants in the fibrin group of the study had 2 mL Hemaseel APR fibrin sealant (Haemacure, Sarasota, FL), which had been mixed intraoperatively and diluted twice according to printed standard instructions and sprayed into their wound cavity by use of an aerosol spray applicator (HemaMyst Surgical Applicator System; Haemacure) immediately before closure of a loose-ladder 4-0 coated Vicryl or 4-0 Monocryl suture line. Gentle, even pressure was then applied to the chest wall for 5 minutes. When axillary dissection was performed, study participants had 2 mL Hemaseel APR fibrin sealant sprayed into the site of axillary dissection also immediately before closure. Therefore, participants received 4 mL fibrin sealant. Participants in both groups of the study underwent placement of 1 or 2 Jackson-Pratt drains in their wound and/or axilla according to the current standard of care. In both groups, dry, light dressings were placed, and arm exercises began on the first postoperative day; lifting >10 pounds or lifting the arm above shoulder level was avoided for 2 days. Participants were followed up by way of routine postoperative clinic visits for 1 month from the date of surgery or, in the case that a seroma developed, until it resolved. Drain removal took place when total drain output was <30 mL/24 hours. Inspection and palpation were used to determine seroma formation, and aspiration was performed as needed. 

Preoperative data collection included age, hospital, surgeon, medical and surgical history (biopsy and surgical history, steroid use, chemotherapy, and radiation therapy history), and comorbidities (smoking, diabetes, renal insufficiency). Surgical data collected included procedure performed, estimated blood loss, number of axillary nodes, number of positive axillary nodes collected, final pathologic diagnosis, cancer stage, and specimen weight. Postoperative data collected included length of hospital stay, postoperative day of drain removal, complications (infection, flap necrosis, hematoma, and open wound), incidence of seroma formation, interval to seroma resolution, seroma aspirate volume, and number of postoperative visits. Seroma formation was defined as inability to remove participant drain by postoperative day number 10 because of high output (drain seroma) and/or the need to aspirate lymphatic fluid from the breast after surgery.

Analysis of qualitative and quantitative data was performed using chi-square analysis and analysis of variance, respectively. Analyses of quantitative variables were evaluated by Bartlett's test for equal variances. If this test identified heterogeneity of variance, the data was subjected to log transformation or the Box-Cox transformation procedure and reanalyzed after heterogeneity of variance had been corrected. If transformation procedures were unsuccessful in correcting heterogeneity of variance, treatment differences were compared using the Wilcoxon rank-sum test.

## 3. Results

8 surgeons operated on a total of 60 women enrolled into the study. The mean age of all participants was 59.1 (S.D: ±12.0 years (range 33 to 78)). All participants had undergone previous FNA or open biopsies. Of the 60 participants, 19 (37.7%) underwent breast conserving surgery plus axillary lymph node dissection and 41(68.3%) underwent modified radical mastectomy. Sentinel lymph node biopsy was not performed in our study due to lack of facility. Pathologic diagnosis was obtained in all of patients. The most common pathologic diagnoses observed were invasive ductal carcinoma (*n* = 33 or 55%) and infiltrating ductal carcinoma (*n* = 27 or 45%). An analysis was carried out to compare these two groups to determine if they were significantly different. Analysis revealed that nearly all measured parameters were similar between these groups. Of these participants, incidence of seroma formation was 16.1% for fibrin glue group and 24.1% for drain group (*P* = 0.204). Participants in the control and fibrin glue groups were well matched with regard to age, weight, and height (Table 1). Within the fibrin glue group, there was a nonsignificant trend toward a higher incidence of diabetes compared with the control group (Table 1). Data regarding the surgical procedures performed are listed in Table 2. Numbers of lymph nodes removed, positive lymph nodes, and tumor size were all similar in participants in the fibrin glue and drain groups. Pathologic type and tumor grade in the drain and fibrin glue groups were similar (Table 3). Data regarding evaluation of complications observed, seroma formation, and interval to seroma resolution are listed in Table 3. Overall complication rate for participants in the drain and fibrin glue groups were similar as were incidences of independent complications (i.e., infection, flap necrosis, hematoma, and open wound). Although there was a trend toward higher incidence of seromas in the drain group compared with the fibrin glue group (24.1% versus 16.1%), this trend was not significant (*P* = 0.43). One half of the participants in the drain group who developed seromas were categorized as having developed a drain seroma. As detailed in Section 2, a drain seroma was defined as prolonged output from the drain >30 mL/d output at >10 days after surgery. Cumulative aspirate volumes for seromas in the drain group fibrin glue group were significantly greater than in the fibrin glue group (210 versus 110 mL; *P* = 0.0015). Seroma formation rate in fibrin glue group was 1/16% and in control group was 1/24%, the difference was not significant. Cumulative seroma volume in fibrin glue and control groups was 110 and 210 mL, respectively. Mean volume aspirated in fibrin glue group was 22.00 ± 2.73 mL and in control group was 30.00 ± 8.16 mL, there was no significant differences.

## 4. Discussion 

There is an extremely high incidence of seroma formation after breast cancer surgery and axillary node dissections, and this incidence ranges from 15% to as high as 60% [18]. Although not life threatening, seromas do account for significant patient expense and morbidity including frequent aspiration, pain, wound dehiscence, infection, prolonged hospitalizations, decreased limb mobility, delayed wound healing, arm swelling, flap necrosis, and reoperation. A review of the literature reveals multiple investigations into the cause and prevention of seromas including attempts at surgical obliteration of dead space [19], intraoperative placement of drains [20], sclerotherapy [21], talc poudrage, shoulder immobilization, and pressure dressings. Some of these techniques show moderate decreases in seroma formation, but all have their own associated complications including increased inflammation, flap necrosis, peripheral nerve damage, and pain. A definitive and yet safe method for seroma decrease in breast cancer surgery has not yet been demonstrated. Two factors influence the formation of seromas: (1) the oozing of small vessels and (2) the creation of a cavity caused by the removal of tissue. An effective surgical adhesive could improve hemostasis and tissue adherence, thereby decreasing the frequency of these postoperative complications. Breast cancer surgery, and in particular axillary lymphadenectomy, has changed in the last few years with the advent of the sentinel lymph node technique. However, even today there are a number of situations where a conventional axillary lymphadenectomy is indicated, including patients with tumors greater than 3 cm in diameter, with positive or suspect axillary lymph nodes based on an objective examination and an instrumental diagnosis, or with a positive sentinel lymph node. In this group of patients, the axillary lymphadenectomy still has complications, in particular seroma formation (15%–81%), [22] which can delay the patient's discharge, healing, and supplementary radiotherapy and chemotherapy treatments. The formation of seroma can result from a lesion of the axillary lymphatic vessels or from an inflammatory reaction, which may also be prolonged and intensified by the continuance of suction drainage. For some surgeons, removal of drains is when the volume per day is less than 50 mL, but we prefer to remove the drains when the volume per day is less than 30 mL, since in our experience drains do not prevent seroma formation, but they do dictate the date of discharge, resulting in a longer stay. A number of surgeons believe that it is possible to discharge patients with the drain in situ [21–23] despite the associated discomfort and increase in percentage infection rate. This issue of drainage management in the home arises in cases of procedures performed in day surgery clinics. Many published articles on the usefulness of drainage following axillary lymphadenectomy contradict each another with regard to seroma control, magnitude, and duration. Somers et al. [24] compared patients with or without drainage and found that there was a greater incidence and degree of seroma, with longer duration, in patients without suction drainage. Porter et al. [25] reported a nonsignificant difference in the incidence and degree of seroma between patients with suction drainage (73%) versus patients without suction drainage (89%). Divino et al. [26] reported a 6% incidence of seroma for patients with drainage, compared with 40% for patients without. Petrek et al. [27] demonstrated a relationship between the magnitude of the seroma and lymph node positivity based on metastasis or earlier lymph node biopsies. Burak et al. [28] noted a relationship between seroma magnitude and patient age. Salmon et al. [29] reported a positive correlation between the incidence of seroma and size of the breast removed. Using ultrasound monitoring, Jeffrey et al. [30] reported a 92% seroma incidence in patients without drains; sharing the view of others that repeated suctions may be the cause of infections of the axillary fossa, [31–34] this group applied evacuative puncture only in symptomatic cases (42%). In terms of the methods of reducing seroma magnitude, there have been numerous reports of the benefits of using an external compression dressing, immobilization of the arm, use of sutures to close the axillary fossa, excessive use of the electric scalpel compared to ligature of the lymphatic branches, benefits of multiple drains, and the type of suction (high or low pressure) applied [31–34] The use of fibrin glue has also produced contrasting results: reduction of seroma according to Moore et al. [35] and Gilly et al. [36] and no difference in seroma formation compared with patients treated without fibrin glue according to Burak et al. [28], Langer et al. [37], and Dinsmore et al. [12]. The latter authors attributed the lack of benefit to the presence of drainage possibly interfering with the stabilization of a fibrin clot and with closure of the lymphatic capillaries. Fibrin glue interacts with the tissues damaged during the surgical procedure, favoring the growth of fibroblasts and wound healing. It favors hemostasis by preventing hematomas, which delay the surgical healing processes, makes the lymphatic branches impermeable, reducing seroma formation, and makes it possible to close the dead spaces through tissue adhesion. A number of papers have presented comparative studies of patients with and without fibrin glue in the axillary fossa. In a study of 24 patients who underwent quadrantectomy or mastectomy with axillary lymphadenectomy, Florio et al. [38] demonstrated that the use of fibrin glue reduced seroma magnitude. In a group of 186 patients, Tasinato et al. [39] reported a reduction in seroma magnitude, duration, and the number of evacuative suctions in patients on whom fibrin glue spray was used, compared with patients whose axillary fossa was only washed with povidone-iodine or saline solution. In a group of 20 patients who underwent quadrantectomy with axillary lymphadenectomy, Tirelli et al. [40] noted a reduction in the magnitude and duration of seromas in patients on whom fibrin glue was used. In a study involving 116 patients who underwent a surgical procedure of quadrantectomy or mastectomy with axillary lymphadenectomy, Jain et al. [41] reported a seroma incidence of approximately 40%, with a reduction in seroma magnitude in mastectomy patients on whom fibrin glue was used. However, a significant difference was not observed between quadrantectomy patients treated with or without fibrin glue. The study demonstrated that suction drainage did not limit the incidence or magnitude of seromas, and that it was associated with extended time spent in hospital and postoperative pain. Yet in a study of 87 patients, Soon et al. [42] showed that, among patients who underwent quadrantectomy or mastectomy with axillary lymphadenectomy, there was no difference in terms of the incidence of seromas with or without the use of suction drainage, and that for the group of patients without drainage, the seromas formed in greater magnitude and for a longer duration, but with a lower percentage of complications.

Based on our experience, and reviewing data from the literature, it seems that the magnitude and duration of the seromas are limited, but they are present in more than 80% of patients, without significant differences between mastectomy and quadrantectomy. The use of fibrin glue in conjunction may therefore be useful in traditional breast cancer surgery for reducing seroma magnitude and duration, and for shortening the stay in hospital, which, in this pathology too, is increasingly conducted in day surgery clinics. Our study was pursued to determine the efficacy of fibrin sealant to decrease postoperative seroma formation after breast surgery. It is believed that fibrin sealant acts by improving hemostasis and tissue adherence. Hemaseel APR fibrin sealant is a topical commercial preparation of fibrinogen and thrombin that contains 3 to 10 times the amount of fibrinogen found in cryoprecipitate. It currently has indications as an adjunct to hemostasis in surgeries involving cardiopulmonary bypass and treatment of splenic injuries caused by blunt or penetrating trauma to the abdomen when control of bleeding by conventional surgical techniques—including suture, ligature, and cautery—is ineffective or impractical. The 2-component fibrin sealant contains the following four substances: (1) sealer protein concentrate (human), (2) fibrinolysis inhibitor solution (bovine), (3) thrombin (human), and (4) calcium chloride solution. Mixing the sealer protein and thrombin solutions produces a viscous solution that quickly sets into an elastic coagulum [43]. Our study showed a lower rate of seroma formation in patients that used fibrin glue plus conventional drain placement (16.1%) versus conventional drain placement (24.1%), but this difference was not significant. Total aspirate volumes were significantly higher in the drain group (*P* < 0.05). 

Patients in the drain group produced seromas with the highest aspirate volumes (210 mL), and 2 patients complained of poor cosmetic results. Originally, 62 participants were planned for this study; however, 2 patients excluded from study due to lack of cooperation of these two patients (from drain group). Other limitation of this study was poor charting by participating surgeons as to aspiration volumes. 

Previous autologous fibrin sealant use during modified radical mastectomy has not resulted in a clinically significant decrease in seroma formation [44], as well as our study. We propose that although fibrin sealant does have a seroma rate comparable with that of drain placement, the higher cost and cumbersome technique tend to indicate that there is no real advantage to using fibrin glue over a drain. Decrease in morbidity and discomfort from the placement of drains would be a real boon to those undergoing these arduous procedures; however, the higher cost and the possibility of poor cosmetic result would tend to negate these advantages. Results of this study show that use of fibrin glue did not significantly prevent seroma formation and had not reduced seroma magnitude and duration; hence, the current evidence does not support the use of fibrin products in breast cancer surgery to reduce postoperative drainage or seroma formation.

 Therefore, we believe that use of fibrin glue is not a viable option to supplant drain placement in breast surgery at this time. Prospective trials will be necessary to asses efficacy of fibrin glue in seroma formation after breast surgeries. 

## Figures and Tables

**Table 1:** Comparison of demographic data and comorbid conditions for control (drain) and fibrin glue groups.

Parameter	Drain		Fibrin glue		*P* value
	Number	Value	Number	Value	
Number of patients	29	—	31	—	—
Age (y)^*◆*^	—	57.5 ± 11.2	—	58.3 ± 10.7	0.7321
Weight (kg)^*◆*^	—	71.7 ± 21.5	—	74.6 ± 32.3	0.1273
Previous biopsy	29	—	31	—	—
Comorbidity diabetes	3	—	2	—	—

^*◆*^Mean ± SD.

**Table 2:** Comparison of performed, tumor size, and axillary lymph node sampling for the drain and fibrin glue groups.

Parameter	Drain		Fibrin glue		*P* value
	Number	Value	Number	Value	
Number of patients	29	—	31	—	—
Procedure					
Modified radical mastectomy	20	69%	21	67.7%	0.9823
Lumpectomy plus axillary node dissection	10	34.5%	9	29%	0.2412
Tumor size (cm)^*◆*^	29	3.1 ± 2.3	31	4.2 ± 3.1	0.2432
Axillary lymph node (number)^*◆*^	29	14.2 ± 3.4	31	14.7 ± 4.7	0.8629
Positive axillary node^*◆*^	29	2.5 ± 3.2	31	1.2 ± 1.8	0.7834

^*◆*^Mean ± SD.

**Table 3:** Comparison of tumor grade, pathology, days to seroma resolution, seroma aspirate volume, and complications for the drain and fibrin glue groups.

Parameter	Drain		Fibrin glue		*P* value
	Number	Value	Number	Value	
Total number of patients	29	—	31	—	—
Tumor grade					
1	4 (13.8%)	—	5 (16.1%)	—	—
2	10 (34.5%)	—	15 (48.39%)	—	—
3	14 (48.3%)	—	11 (35.5%)	—	—
4	1 (3.4%)	—	0 (0.0)	—	0.623
Tumor pathology					
Invasive ductal carcinoma	15 (51.7%)	—	18 (58.1%)	—	—
Infiltrating ductal carcinoma	14 (48.3%)	—	13 (41.9%)	—	0.531
Complications					
Infection	1 (3.4%)	—	2 (6.4%)	—	0.785
Flap necrosis	0	—	0	—	—
Hematoma	2 (6.9%)	—	2 (6.4%)	—	0.765
Open wound	0	—	0	—	—
Seroma	7 (24.1%)	—	5 (16.1%)	—	0.43
Day seroma resolved^*◆*^	—	18 ± 8.6	—	17 ± 8.9	0.0968
Aspirate volume (mL)^*◆*^	—	210	—	110	0.0015

^*◆*^Mean ± SD.
